# Experimental investigation of the effects of water content on the anisotropy of mode I fracture toughness of bedded mudstones

**DOI:** 10.1371/journal.pone.0237909

**Published:** 2020-08-27

**Authors:** Jianfeng Yang, Li Li, Haojie Lian

**Affiliations:** 1 School of Energy Engineering, Xi’an University of Science and Technology, Xi’an, Shaanxi, China; 2 Key Laboratory of In-situ Property-Improving Mining of Ministry of Education, Taiyuan University of Technology, Taiyuan, Shanxi, China; 3 College of Mining Engineering, Taiyuan University of Technology, Taiyuan, Shanxi, China; 4 Civil and Environmental Engineering, University of Waterloo, Ontario, Waterloo, Canada; China University of Mining and Technology, CHINA

## Abstract

The influence of water content on mode I fracture toughness (*K*_Ic_) of mudstones has been studied using semi-circular bend (SCB) specimens subject to three-point bendings. And the mudstone SCB specimens are divided into three types, including Type-A, Type-B and Type-C, corresponding to the three configurations of the bedding planes, including divider direction, arrester direction, and transverse direction, respectively. The test results show that the values of *K*_Ic_ for the three types of specimens are different due to the bedding structure, the Type-A specimens have the largest value of *K*_Ic_ for the same soak period, while the Type-C specimens possess the smallest value. As the soak period increases, the *K*_Ic_ of the three kinds of mudstone specimens decreases, and the fracture mechanisms of the specimens change gradually from the brittle failure form to the ductile failure form. Moreover, the standard deviation was used to quantify the anisotropy degree of the *K*_Ic_ of the mudstone samples. As the water content increases, the standard deviation increases from 0.057 to 0.139, which indicates a significant increase in anisotropy of the *K*_Ic_ of the mudstone specimens. In addition, the acoustic emission (AE) system was used to detect the AE events associated with the fracture initiation and propagation in the mudstone specimens for the different water content, with the raising water content, the cumulative AE events decrease, and the standard deviation of AE events increases, repesenting that the anisotropy of the AE events of the three types of specimens becomes more prominent. Further, the relationship between the tensile strength (*σ*_t_) and the *K*_Ic_ of the three types of mudstone specimens for different water contents has been proved to be the linear relation.

## Introduction

Mudstones widely exist in various engineering geological environments, such as mining [1], hydraulic fracturing [2] and tunnelling. As a type of sedimentary rock, mudstones contain bedding structures, which can induce the anisotropy of the physical and mechanical properties along different bedding planes. Sayers [3] investigated the effect of bedding orientation on the Young’s moduli and Poisson’s ratios of clay shales, and the experiment result shows that the two values on the direction vertical to the bedding plane are obviously different from those on the direction parallel to the bedding plane. Zhou et al. [4] tested the thermal expansion and the P-wave velocity of sedimentary rocks along the different bedding directions, and these physical parameters showed significant anisotropy. Liu et al. [5] confirmed that the anisotropy of the static and dynamic uniaxial compression strength exists in bedded rocks. Claessona and Bohloli [6] analyzed the stress field and the tensile strength of anisotropic rocks with bedding by using an analytical solution. Ghazvinian et al.’s [7] experimental research shows that the shear strength is anisotropic because of the existence of the bedding plane in rocks.

Under natural and geological engineering conditions, the water content is a important factor that affects the mechanical and physical characteristics of the argillaceous rocks and enhances the anisotropy of rocks. Vergara and Triantafyllidis [8] carried out triaxial tests to study the influence of water on the mudstone mechanical properties, and the test results indicate a strong decrease of strength and stiffness with increasing water saturation. Li et al. [9] found that the shear strength of mudstones decreased obviously along with the increasing moisture content. Erguler et al. [10] and Pham et al. [11] reached a similar conclusion for mudstones through mechanical experiments. Vales et al. [12] investigated the effect of water content on anisotropic mechanical features, including cohesion, elastic modulus, uniaxial compressive strength and triaxial compressive strength, of mudstones, finding that the anisotropy increased significantly with the increase of water content. Karakul and Ulusay’s [13] experimental research found that the wave velocities in mudstones show obviously anisotropic along the different bedding directions with rock-water interaction. Thus the water content plays a key role in increasing anisotropy of mudstones.

Central to the rock engineering is prediction of crack initiation and propagation. As a key parameter in fracture mechanics. Under the background of linear elastic fracture mechanics (LEFM), the stress intensity factor (SIF) was defined by Irwin [14] to describe the stress field distribution near crack tips. The critical value of SIF relating to crack initiation is known as the fracture toughness [15, 16], which is an important mechanical parameter that identify the resistance of the rock against the crack propagation. Since rock is most prone to tensile fracture, mode I (tension mode) fracture toughness is important parameter. And the anisotropy of mode I (tension mode) fracture toughness in bedded rocks is of particular interest. Nara and Kaneko [17] studied experimentally the crack propagation behaviour in anisotropic rock. Chandler et al. [18] measured the mode I fracture toughness of shales and the significant anisotropy is observed in shale fracture toughness measurements. Nasseri and Mohanty [19] and Dai and Xia [20] drew out similar conclusions for granites. Kataoka et al. [21] tested systematically the fracture toughness of anisotropic rocks by semi-circular bend (SCB) tests. Moreover, the reaction of water with mudstones can further enhance the anisotropy [22, 23] as the clay minerals in mudstones cause uneven swelling deformation. To date, little has been reported on the mode I fracture toughness (*K*_Ic_) of mudstones with bedding and the effect of water content on the anisotropy of the *K*_Ic_.

In this research, the mudstone specimens are divided into three types, including Type-A mudstone specimens, Type-B mudstone specimens and Type-C mudstone specimens, based on the orientation of the bedding planes (divider, arrester and transverse, respectively). We performed experimental research on the anisotropy of the *K*_Ic_ of mudstone specimens with the different water contents. Moreover, the acoustic emission (AE) technology was used to monitor the crack propagation behavior of the three kinds of mudstone specimens. Furthermore, the relationships between tensile strength (*σ*_t_) and *K*_Ic_ of the mudstone specimens were investigated.

## Experimental methods and progress

### SCB specimens under three-point bendings

**Fig 1:**
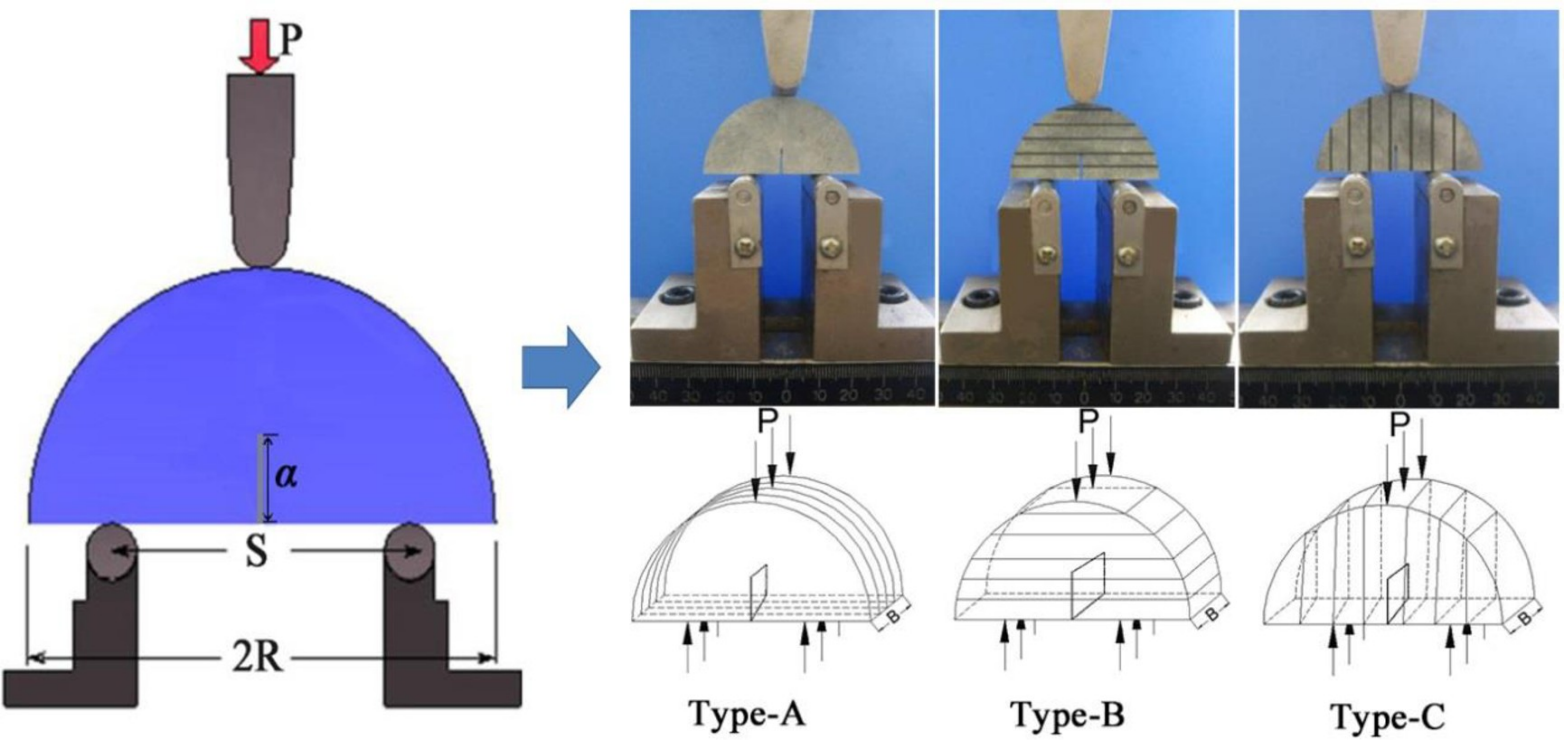
The three types of SCB specimens.

Numerous experimental methods and specimen types have been designed to test the *K*_Ic_ of rocks, such as the diametral compression specimen with a central crack on the discs [24], the cracked chevron notched Brazilian disk specimen [25, 26] and the semi-circular specimens under three-point bending (SCB) [27, 28]. Among them, the SCB specimen is specifically favourable for its simple machining process, the convenient operation procedure, and the minimal use of rock materials. The use of an SCB specimen for testing *K*_Ic_ of the various rocks was first suggested by Chong and Kuruppu [29]. As depicted in Fig 1, the specimen is a semicircular disc with a pre-fabricated crack, which the radius is *R* and the length of the precrack is *α*. Kuruppu et al. [27] suggested using a SCB specimen to conduct *K*_Ic_ tests on rock samples, the *K*_Ic_ of the rock SCB specimen is written as:

$$K_{Ic}=Y^{'}\frac{P_{max}\sqrt{\pi a}}{2RB}$$ (1)

$$Y^{'}=-1.297+9.516(S/2R)-(0.47+16.457(S/2R))\beta +(1.071+34.401(S/2R))\beta^{2}$$ (2)

where *B* is the thickness of the mudstone SCB specimen; *P*_max_ is the peak force; *β* is equal to the rate of the precrack length (*α*) to the SCB specimen radius (*R*); and *S* is the distance between the center of two roller supports. In Eq (2), *Y*^*’*^ is the mode I geometry factor and depends on the geometric parameters *S/*2*R* and *α/R*. *Y*^*’*^ can be derived by using the finite element method.

### SCB specimen preparation and test progress

**Fig 2:**
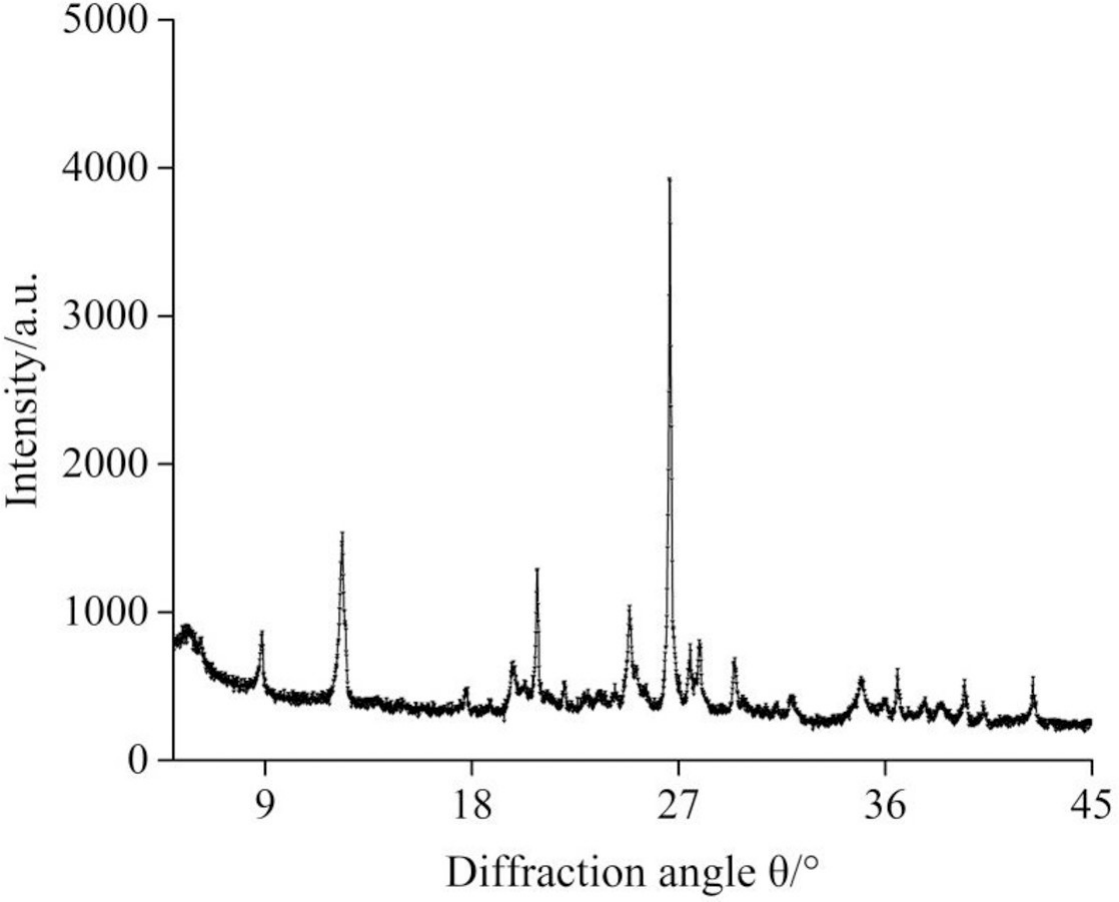
XRD spectrum of the mudstone.

The mudstone samples tested in this study were extracted from the roof strata of No. 8 coal seam at the Xiegou Colliery in Shanxi Province, China. The mineral crystal composition of the mudstone was determined by using X-ray diffraction (XRD) and the typical XRD schema is shown in Fig 2. The content of clay minerals in the specimens is 34%, which mainly includes kaolinite and illite. The rest is composed mainly of silicon dioxide and potash feldspar. It is difficult to acquire mudstone specimens using traditional drilling methods, because of the bedding architecture, the high probability of mechanical damage and the affection of water. To solve this problem, in this work the core samples were obtained using a carborundum wire saw with the diameter of 1.5 mm in a numerically controlled machine tool with lubricating oil coolant. First, we obtained *Φ*50 mm×50 mm cylinder mudstone specimens with the bedding planes perpendicular and parallel to the axis direction, respectively. Then, we performed experiments on the water content, absolute swelling ratio and P-wave velocity of the specimens after being soaked for 0, 50, 100, 200 and 300 minutes in the water at atmospheric pressure. The results are given in Table 1.

**Table 1:** Average water content, absolute swelling ratio and P-wave velocity of the mudstone after being soaked for different lengths of time.

soaking time	average water content	absolute swelling ratio ψ(%)		P-wave velocity V_p_ (km/s)	
T(mins)	*w* (%)	perpendicular	parallel	perpendicular	parallel
0	0	0	0	2.85±0.02	2.89±0.02
50	1.9	0.97	0.24	2.33±0.02	2.74±0.02
100	3.2	1.54	0.32	2.07±0.02	2.68±0.02
200	6.0	2.98	0.61	1.63±0.02	2.43±0.02
300	8.1	3.52	0.75	1.32±0.02	2.31±0.02

Table 1 shows that the mudstone is anisotropic after being soaked in water for different time period. As the average gravimetric water content increased, the mudstone showed obvious anisotropy of absolute swelling ratio and P-wave velocity. Therefore, following the suggestion of ISRM [27], we defined three types of mudstone SCB specimens, which are assigned the Type-A (divider) SCB specimen, the Type-B (arrester) SCB specimen and the Type-C (transverse) SCB specimen, corresponding to the angle between the pre-fabricated crack and the bedding direction (Fig 1). Consequently, the semi-circular specimens were cut into pieces with a radius of 25 mm (*R* = 25 mm) and the thickness of 20 mm (*B* = 20 mm). Afterwards, a straight notch was prefabricated for each semi-circular specimen, and the ratio *β* was set to 0.4.

All the samples were placed in a drying oven at 105°C for 24 h and then cooled to room temperature to preclude the influence of the initial water content. The mudstone samples were then soaked in water for 0, 50, 100, 200 and 300 minutes, and the average water contents reached 0, 1.9%, 3.2%, 6.0% and 8.1%, respectively. In this experiment, the ratio *S/*2*R* was set to 0.5, and the experimental loading rate was set to 0.02 mm/min. At least five mudstone SCB specimens were carried out under each condition.

## Results and discussion

### The *K*_Ic_ of the mudstone SCB specimens

The *K*_Ic_ of the three types of mudstone SCB specimens with the different water contents is shown in Tables 2–4. For the initial mudstone specimens, the average value of the *K*_Ic_ was 0.852 MPa·m^0.5^, 0.823 MPa·m^0.5^ and 0.725 MPa·m^0.5^ corresponding to the Type-A mudstone SCB specimen, the Type-B mudstone SCB specimen and the Type-C mudstone SCB specimen, respectively. As the water content increases, the values of the *K*_Ic_ for the three type specimens constantly decreased. When the water content reached 1.9%, 3.2%, 6.0% and 8.1%, the *K*_Ic_ of the Type-A mudstone SCB specimen reduced by up to 8.10%, 18.90%, 28.96% and 37.18%, respectively, the *K*_Ic_ of the Type-B mudstone SCB specimen reduced by up to 9.33%, 24.54%, 33.90% and 45.44%, and the *K*_Ic_ of the Type-C mudstone SCB specimen decreased by up to 12.83%, 31.59%, 57.66% and 70.90%, respectively. The reduction degrees of the *K*_Ic_ for the Type-C mudstone SCB specimen are higher than that of two other types of specimens, indicating that the *K*_Ic_ of the Type-C mudstone SCB specimen is more sensitive to the effect of water than that of other types of specimens.

**Table 2:** The *K*_Ic_ and the *σ*_t_ of the Type-A mudstone samples.

*w* (%)	Specimen number	*P*_*max*_ (N)	*K*_Ic_ (MPa·m^0.5^)		Reduction Degrees	*σ*_t_ (MPa)	
			test value average value			test value	test value
0	A0-1	1650.8	0.85			4.896	
	A0-2	1598.4	0.823			4.759	
	A0-3	1703.3	0.877	0.852	0	5.029	4.916
	A0-4	1685.7	0.868			4.732	
	A0-5	1635.3	0.842			5.163	
1.9	A50-1	1520.7	0.783			4.672	
	A50-2	1479.9	0.762			4.370	
	A50-3	1543.0	0.795	0.783	-8.10%	4.812	4.636
	A50-4	1545.9	0.796			4.417	
	A50-5	1512.9	0.779			4.909	
3.2	A100-1	1386.1	0.7137			4.410	
	A100-2	1407.1	0.7245			4.344	
	A100-3	1227.0	0.6318	0.691	-18.90%	3.942	4.257
	A100-4	1248.4	0.6428			3.816	
	A100-5	1435.6	0.7392			4.773	
6.0	A200-1	1170.9	0.6029			4.060	
	A200-2	1122.4	0.5779			3.911	
	A200-3	1231.7	0.6342	0.6053	-28.96%	4.013	4.023
	A200-4	1222.9	0.6297			3.885	
	A200-5	1129.9	0.5818			4.245	
8.1	A300-1	1017.1	0.5237			3.662	
	A300-2	1093.0	0.5628			3.882	
	A300-3	1007.0	0.5185	0.5352	-37.18%	3.865	3.823
	A300-4	1004.5	0.5172			3.715	
	A300-5	1075.5	0.5538			3.991	

**Table 3:** The *K*_Ic_ and the *σ*_t_ of the Type-B mudstone samples.

*w* (%)	Specimen number	*P*_*max*_ (N)	*K*_Ic_ (MPa·m^0.5^)		Reduction Degrees	*σ*_t_ (MPa)	
			test value average value			test value	test value
0	B0-1	1586.7	0.817			4.659	
	B0-2	1650.8	0.85			4.836	
	B0-3	1540.1	0.793	0.823	0	4.384	4.611
	B0-4	1575.4	0.8112			4.462	
	B0-5	1638.7	0.8438			4.705	
1.9	B50-1	1439.1	0.741			4.345	
	B50-2	1353.7	0.697			4.074	
	B50-3	1547.9	0.797	0.746	-9.33%	4.516	4.296
	B50-4	1521.1	0.7832			4.106	
	B50-5	1376.6	0.7088			4.438	
3.2	B100-1	1194.4	0.615			3.745	
	B100-2	1243.0	0.64			3.901	
	B100-3	1192.5	0.614	0.621	-24.54%	3.506	3.701
	B100-4	1209.0	0.6225			3.525	
	B100-5	1191.5	0.6135			3.829	
6.0	B200-1	1046.8	0.539			3.350	
	B200-2	1095.4	0.564			3.501	
	B200-3	1050.7	0.541	0.544	-33.90%	3.455	3.417
	B200-4	1075.2	0.5536			3.316	
	B200-5	1014.6	0.5224			3.464	
8.1	A300-1	1017.1	0.5237			2.848	
	A300-2	1093.0	0.5628			3.116	
	A300-3	1007.0	0.5185	0.449	-45.44%	2.835	2.918
	A300-4	910.5	0.4688			2.787	
	A300-5	816.1	0.4202			3.004	

**Table 4:** The *K*_Ic_ and the *σ*_t_ of the Type-C mudstone samples.

*w* (%)	Specimen number	*P*_*max*_ (N)	*K*_Ic_ (MPa·m^0.5^)		Reduction Degrees	*σ*_t_ (MPa)	
			test value average value			test value	test value
0	C0-1	1536.2	0.791			4.877	
	C0-2	1335.0	0.6874			4.217	
	C0-3	1382.2	0.7117	0.725	0	4.229	4.419
	C0-4	1469.8	0.7568			4.283	
	C0-5	1317.1	0.6782			4.489	
1.9	C50-1	1192.9	0.6142			3.941	
	C50-2	1156.7	0.5956			3.817	
	C50-3	1379.3	0.7102	0.632	-12.83%	4.427	3.926
	C50-4	1375.4	0.7082			3.852	
	C50-5	1032.8	0.5318			3.589	
3.2	C100-1	967.0	0.4979			3.280	
	C100-2	1065.6	0.5487			3.629	
	C100-3	927.6	0.4776	0.496	-31.59%	3.226	3.392
	C100-4	860.0	0.4428			3.257	
	C100-5	996.7	0.5132			3.569	
6.0	C200-1	672.2	0.3461			2.560	
	C200-2	601.3	0.3096			2.282	
	C200-3	561.7	0.2892	0.307	-57.66%	2.216	2.337
	C200-4	540.3	0.2782			2.301	
	C200-5	605.5	0.3118			2.322	
8.1	C300-1	426.1	0.2194			1.960	
	C300-2	449.8	0.2316			2.096	
	C300-3	370.9	0.1910	0.211	-70.90%	1.603	1.898
	C300-2	364.7	0.1878			1.786	
	C300-3	437.4	0.2252			2.046	

**Fig 3:**
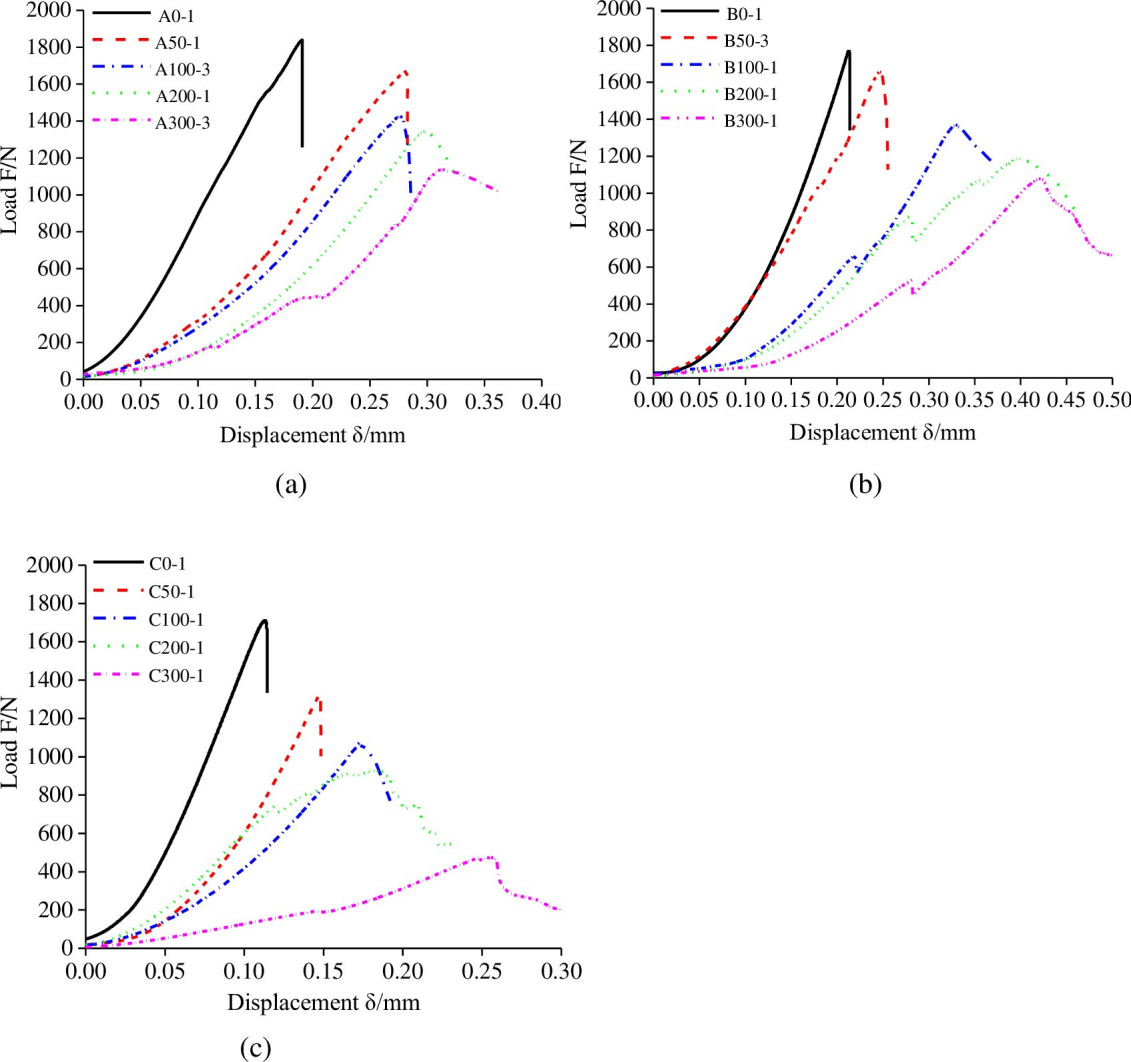
Load–displacement curves of the three types of mudstone specimens for different water contents: (a) the Type-A mudstone SCB specimens, (b) the Type-B mudstone SCB specimens and (c) the Type-C mudstone SCB specimens.

Fig 3 shows the load-displacement curves of the three types of mudstone SCB specimens after being immersed into water for different time periods. The peak loads of the mudstone SCB specimens after being soaked in water were smaller than that of intact mudstone samples, and the peak load of the mudstone specimens decreases with the water content increasing. It indicates that the uneven expansion of the mudstone specimens owing to water results in damage of the rock matrix and reduces the value of *K*_Ic_. The degradation mechanisms of water on mudstone are detailed in the final section. In addition, the peak load in the Type-C specimens is always less than that in two other type specimens under each water content, implying that the crack growth is easier to be caused when the preset crack is parallel to the bedding. The load vs displacement curve is concave in the initial stage, which can be ascribed to the closure of internal microcracks under the loading force. In the elastic stage, the curves reveal a linear relationship between loads and displacements until the peak load is reached. As the water content increases, the compaction stage becomes wider, meanwhile, the slope of the load vs displacement curves in the linear elastic stage observably reduces. This degradation is more obvious in the Type-C (transverse) mudstone specimens than in the other two types, this is because that the strength degradation of the bedding structure due to the influence of water could reduce crack propagation resistance in Type-C SCB specimens. Moreover, the brittle fracture occurs in the mudstone specimens in the initial state or with low water content. However, when the average water content reaches 8.1%, the load did not drop abruptly after the peak (see Fig 3), it indicates that the energy does not dissipate completely after crack propagation in specimens and all the three types of SCB specimens become more ductile. Thus, water influences not only the anisotropy but also the failure characteristics of the mudstones, which change from being brittle to being ductile.

**Fig 4:**
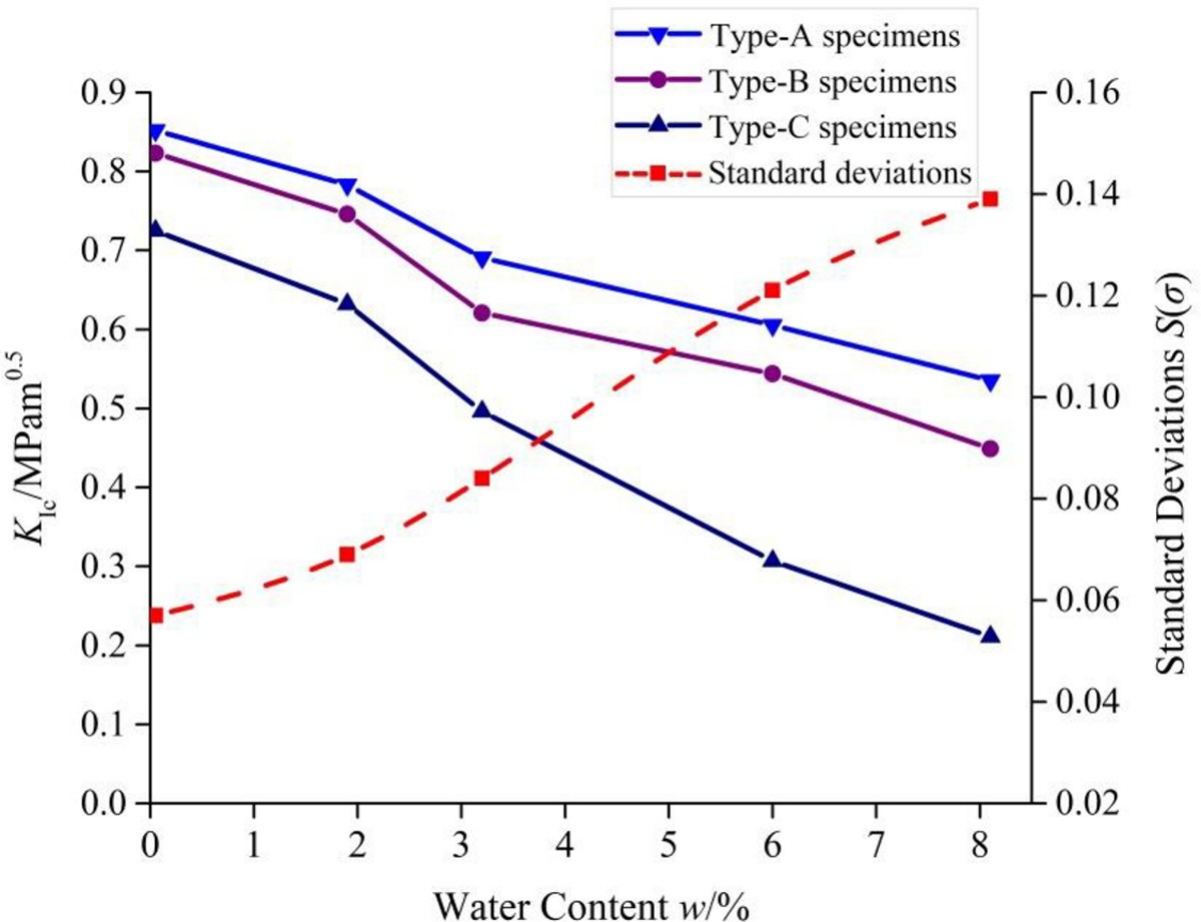
Anisotropy of the *K*_Ic_ of the three types of the mudstone specimens.

The variations of the *K*_Ic_ of the three types of mudstone specimens were different. For each water content, the anisotropy of the *K*_Ic_ was represented by the standard deviation *S*(*σ*), which is written as follows:

$$S\left(\sigma \right)=\sqrt{\frac{1}{N}\sum_{i=1}^{N}{\left(x_{i}-\mu \right)}^{2}}$$ (3)

where *x*_*i*_ denotes different experimental results of the specimens with the given water content, *μ* is the average test result under the same water content condition, and *N* is the number of specimens. The standard deviation value will increase as the degree of the test data scatter increases. As seen in Fig 4, for the initial mudstone samples without being soaked in water, the standard deviation of the three types of mudstone specimens is 0.057. As the water content increases, the value of the standard deviation increases gradually, when the water content reaches 8.1%, the standard deviation of the mudstone specimens with bedding increases to 0.139, suggesting that the anisotropy of the *K*_Ic_ of the three types of specimens becomes more pronounced with the increase of water content.

#### Acoustic emission (AE) results of the mudstone SCB specimens.

**Fig 5:**
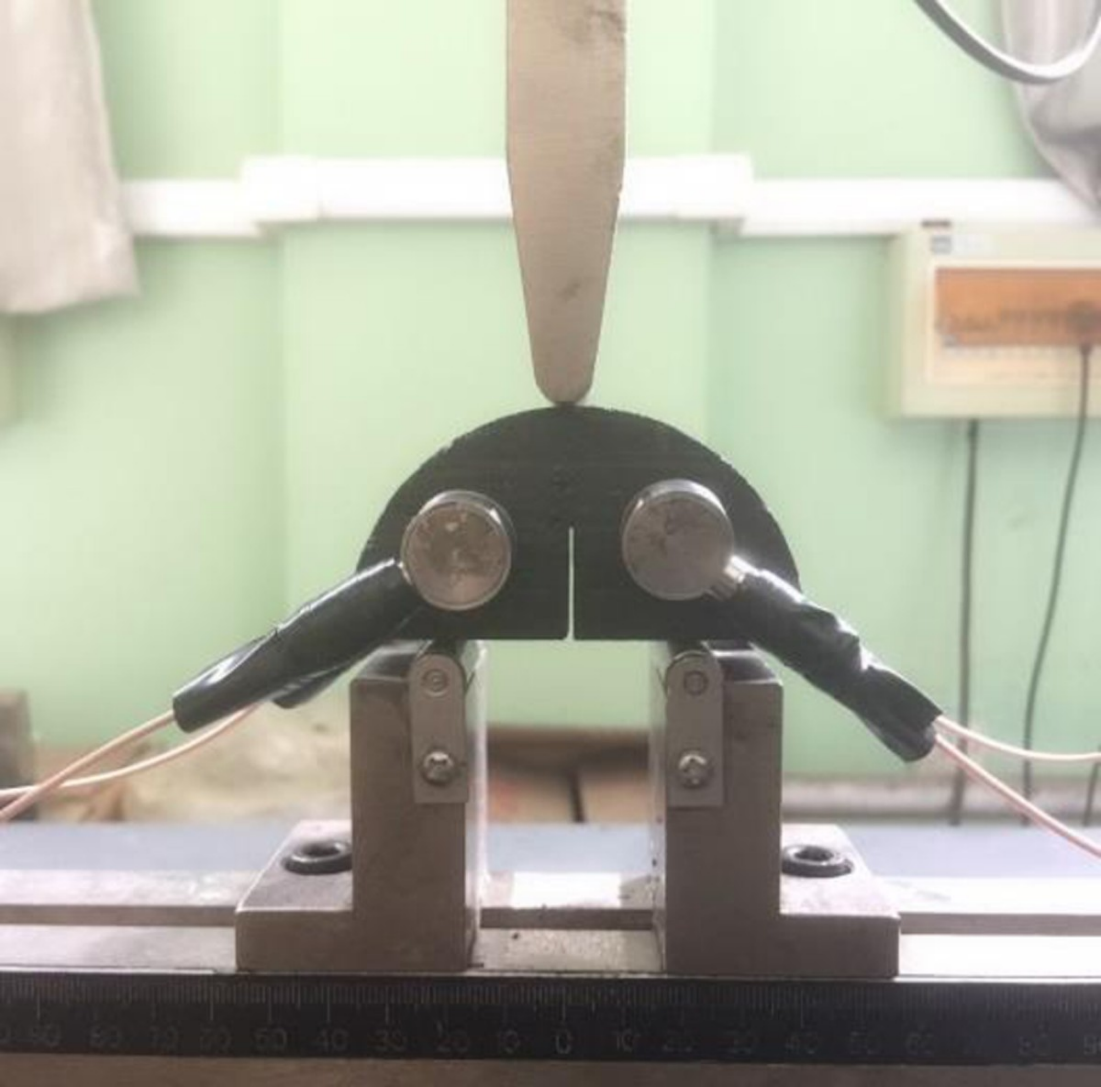
The mode I SCB test with AE monitoring.

**Fig 6:**
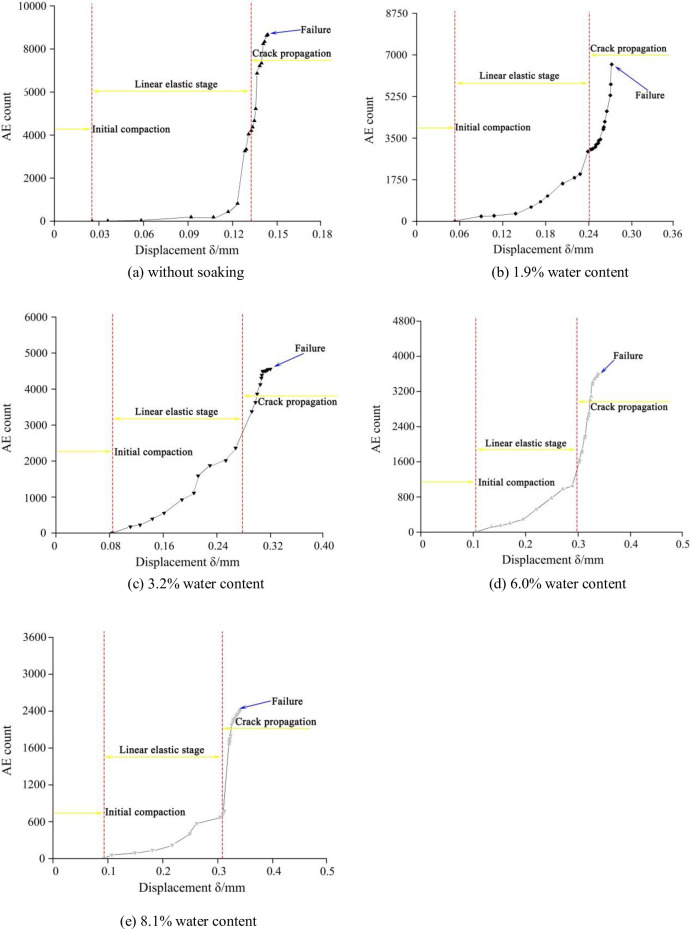
AE count vs displacement curves of the type-A specimens under different water content conditions, including: (a) without soaking, (b) 1.9% water content, (c) 3.2% water content, (d) 6.0% water content, (e) 8.1% water content.

**Fig 7:**
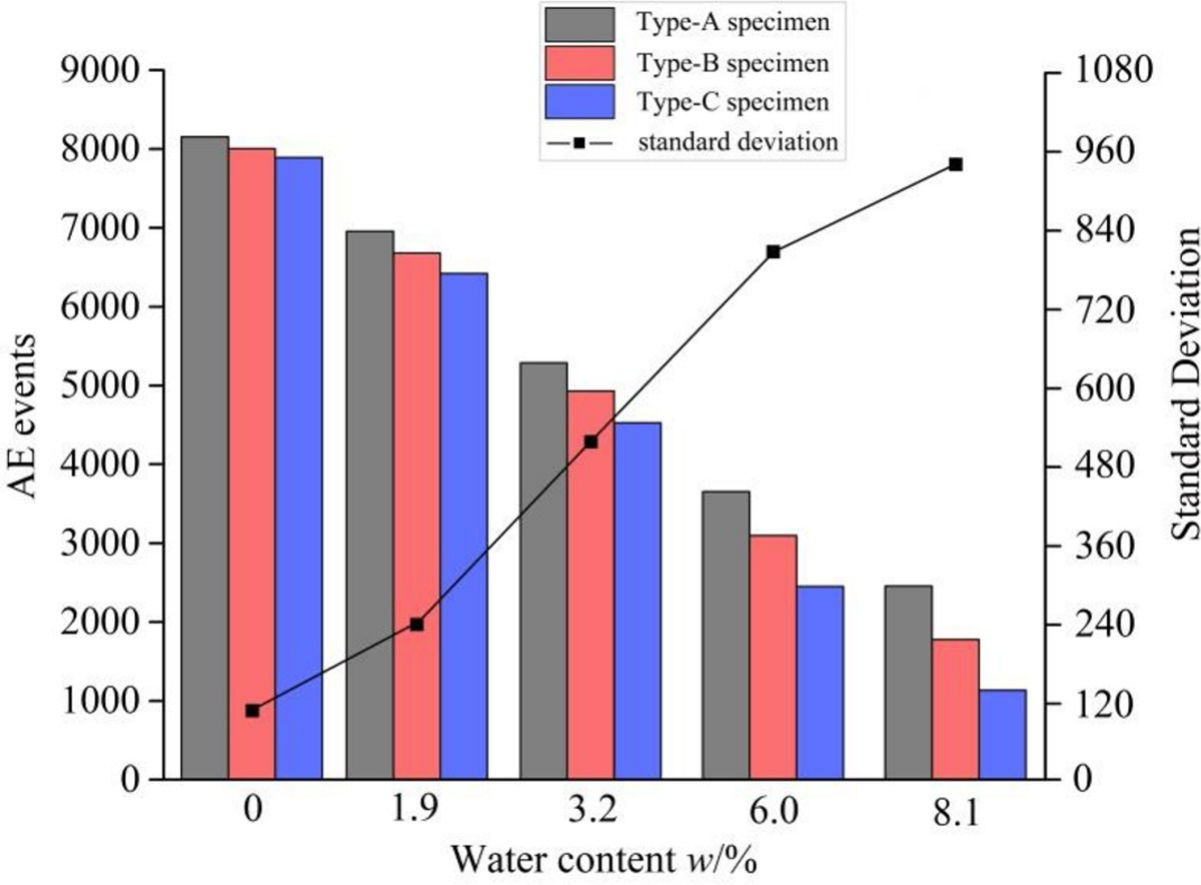
The cumulative AE events of the three types of mudstone specimens under different water content conditions.

The AE technology has been applied to identify the release of energy in the rock mass during the fracture initiation and propagation by counting acoustic emission events [30, 31]. And the increase in AE events indicates that the new crack formation in the solid material needs additional work of the external force. During the mode I loading for the three types of mudstone specimens, PCI-2 acoustic emission (AE) system was used to detect the AE events associated with the fracture initiation and propagation in the mudstone specimens. In this test, four R3*α* acoustic emission sensors were positioned on the two sides of the symmetry axis of the SCB specimen, and the transmission gain of the preamplifiers was set as 25 dB. During the SCB test, the acoustic emission monitoring should keep pace with the force loading. And the mode I SCB tests with AE monitoring are shown in Fig 5. Ultimately, two SCB mudstone specimens were tested under each set of the water content conditions. According to Fig 6, take the Type-A mudstone SCB specimens for example, three fracture propagation periods can be identified by means of the AE technology: initial compaction, linear elasticity, and crack propagation. In the initial compaction stage, the micro-cracks or micro-voids in the coals close under the external force and almost no AE events can be counted. A continuous increase in the cumulative number of AE events takes place with increasing the axial force during the linear elastic stage. In particular, the number of AE events increases sharply when the applied force is approaching the peak strength (crack propagation period). Thus, the number of cumulative AE events reaches its maximum just prior to the coal specimen failure. Compared with the original samples, the cumulative AE events of the water-saturated samples decrease. As shown in Fig 7, for the three types of mudstone samples, the cumulative AE events were recorded during the whole experiment process. For the original mudstone specimens, the average cumulative number of AE events is 8156, 8005 and 7892 corresponding to the Type-A mudstone SCB specimen, the Type-B mudstone SCB specimen and the Type-C mudstone SCB specimen, respectively. And the Type-C mudstone specimen has the least number of AE events, it indicates that the fracture in the mudstone specimen along the bedding direction will consume less energy. With the increase of water content, the cumulative AE events gradually decrease, when the moisture content reaches 8.1%, the cumulative AE events decrease to 2456, 1778 and 1136 corresponding to the Type-A mudstone SCB specimen, the Type-B mudstone SCB specimen and the Type-C mudstone SCB specimen, respectively. It can be expected that the water-saturated samples had already developed internal fractures due to the adsorption swelling, and the developed cracks were utilized for the mudstone sample failure which caused the reduction of the released energy, leading to the decline in AE events. Moreover, the standard deviation of AE events increases with the raising water content, when the water content rises from 0 to 8.1%, the standard deviation increases from 108.15 to 933.50 (see Fig 7). It means that the anisotropy of the AE events for the three types of specimens becomes more prominent with the increase of water content.

### Relationship between the *σ*_t_ and the *K*_Ic_ of the mudstone specimens

Tensile fracture always occurs because of extension of a mode I crack, and the fracture surfaces of two forms of destruction are often similar. Therefore, Zhang [32] suggested that the *K*_Ic_ and the *σ*_t_ of rocks should be related to each other under quasi-static loading. In this test, the Brazilian splitting method [33] was used to measure the *σ*_t_ of a mudstone after being soaked for the same time as the SCB specimens. The Brazilian specimens were also divided into three types according to the direction of the bedding, analogous to the Type-A, Type-B and Type-C in SCB samples. The specimens had a diameter of 50 mm and a thickness of 25 mm. The experimental results are shown in Tables 2–4. As the water content increases, the tensile strength decreases, as the change of the *K*_Ic_ toughness of the mudstone.

**Fig 8:**
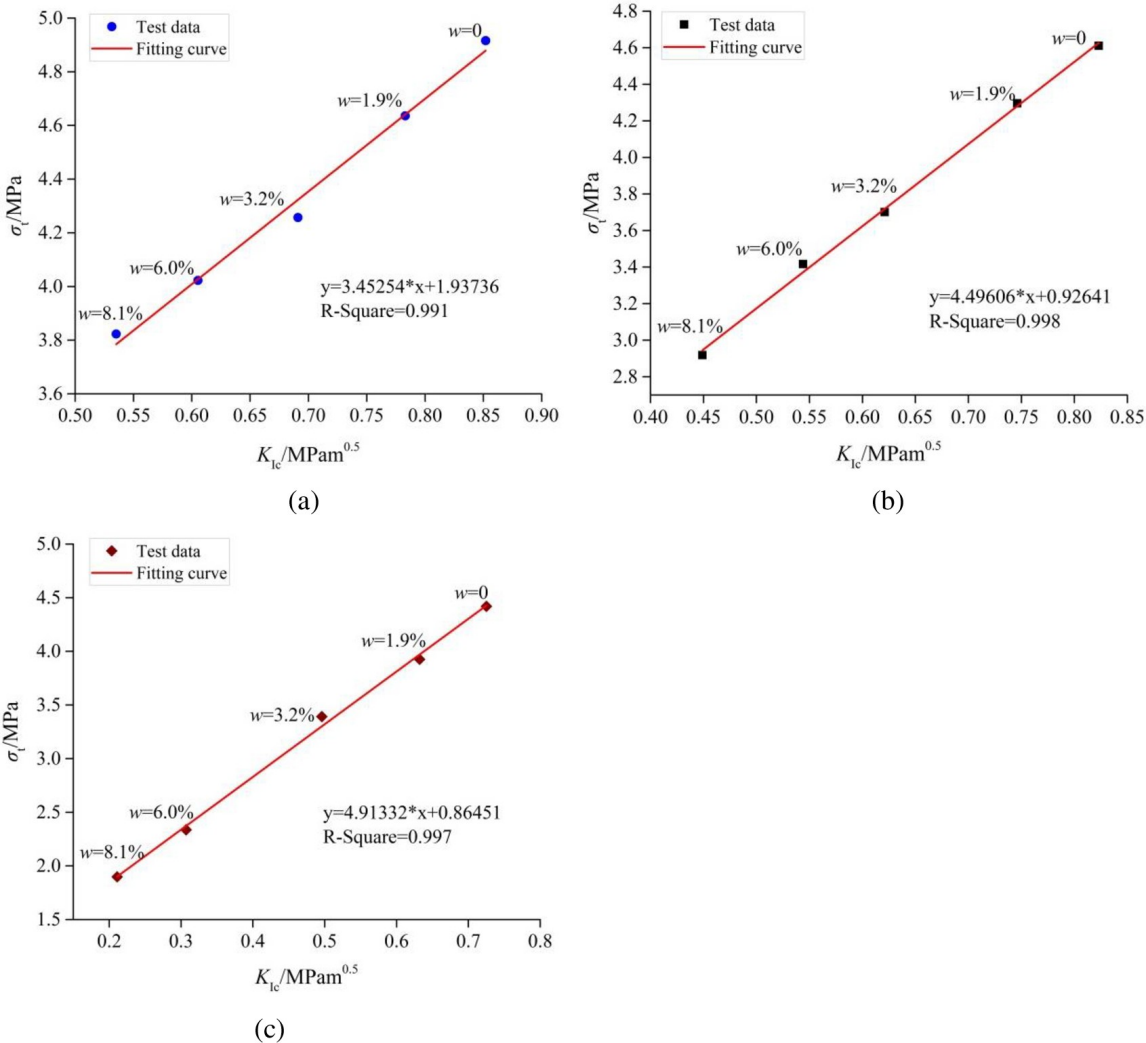
Relationship between the *σ*_t_ and the *K*_Ic_ of the mudstone specimens: (a) the Type-A mudstone specimens, (b) the Type-B mudstone specimens and (c) the Type-C mudstone specimens.

Many investigators [34, 35] obtained a consistent conclusion that the correlation between the *σ*_t_ and the *K*_Ic_ of the various rocks is a linear correlation. According to the experimental results in this research, the relationship between *σ*_t_ and *K*_Ic_ of the three types of mudstone specimens for different water content is shown in Fig 8. A good linear relationship between *σ*_t_ and *K*_Ic_ of the three types of mudstone specimens exists. The fitted equations are *σt* = 3.45254*K*_*Ic*_ + 1.93736, *σt* = 4.49606*K*_*Ic*_ + 0.92641 and *σt* = 4.91332*K*_*Ic*_ + 0.86451 for the Type-A mudstone specimens, the Type-B mudstone specimens and the Type-C mudstone specimens, respectively. The three equations are remarkably different from each other, and also differ from the results provided by Zhang [32]. The differences stem from the bedding structures of mudstones, and the anisotropy was enhanced by the effect of water.

### Influence of water on a mudstone with bedding

Gibbs’ and Griffith’s theories can be used to explain the deterioration mechanism of mudstone with the influence of water, and these theories are expressed in the following formulas.

$$d\gamma =-\sum_{i}\left(\Gamma_{i}d\mu_{i}\right)$$ (4)

$$\sigma =\sqrt{\frac{2\gamma Ε}{\pi \alpha }}$$ (5)

where *γ* is defined as the surface energy of unit length crack in solid materials by Gibbs [36], d*μ*_*i*_ is the change in the chemical potential per unit and *Γ*_*i*_ is the surface concentration. And the relationship between *γ* and the tensile stress (*σ*), the has been given in Eq (5) by Griffith [37], in which *E* is Young’s modulus of the material and *α* is the half crack length. According to these theories, the surface energy of mudstones can be reduced because of the replacement of the water-intrusion, and the tensile strength of the mudstones decreases because of the decline of surface energy which could trigger the formation of a new crack surface. Meanwhile, the volumetric swelling of mudstone specimens can be induced by the saturation of water. And the uneven swelling of the mudstone yields non-uniform stress distribution, resulting in the growth of interior micro-cracks.

**Fig 9:**
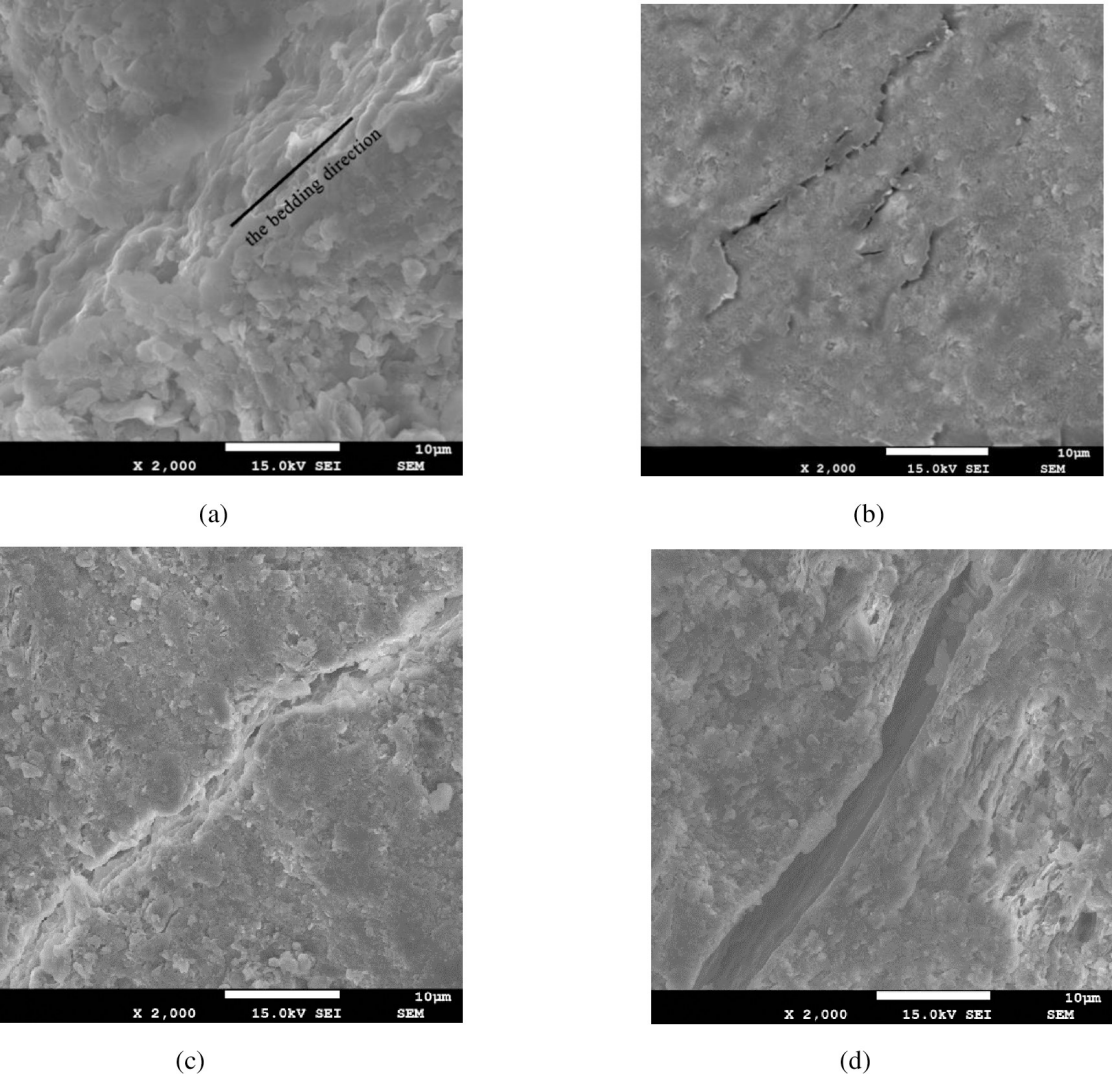
SEM micrographs of mudstone specimens (×2,000) for (a) the intact specimen, (b) the specimen soaked for 100 minutes in water, (c) the specimen soaked for 300 minutes in water and (d) the specimen soaked for far more than 300 minutes.

To give insights into the effect of water on the bedded mudstones in microscopic level, the scanning electron microscopy (SEM) was used to scan the surfaces of mudstone specimens after being soaked in water for different time. Fig 9 display the micrographs at 2,000X magnification for the intact specimen, the specimen after being soaked in water for 100 and 300 minutes, and the specimen after being soaked much longer than 300 minutes, respectively. From Fig 9A, the layered arrangements of the mineral crystal can be seen clearly. And the micro-cracks appears in mudstone specimen for soaking 100 minutes in water (see Fig 9B). In Fig 9C, a micro-crack appears along the direction of the bedding for the specimens after being soaked 300 minutes in the water. The crack is further developed after being soaked far more than 300 minutes (see Fig 9D).

During contact with water, due to the presence of clay mineral particles which exhibit strong hydrophilicity, a water molecular layer can be formed, which continuously extends as the water permeates into these particles. Besides, because the water molecules enter into the interior of the clay mineral crystal, the clay mineral crystal cell layers results in the growth of the spacing and the reduction of the adhesion between the crystals. Ultimately, these reactions cause the growth and expansion of internal micro-cracks, making the *K*_Ic_ values of the mudstone specimens decrease.

## Conclusions

In this test, the effect of water content on anisotropy of the *K*_Ic_ have been studied using SCB specimens, which the mudstone SCB specimens are categorized as three types corresponding to three bedding direction. The experimental conclusions are summarized as follows: As the soaking time increases, the *K*_Ic_ of three types of mudstone SCB specimens decreases, meanwhile the failure features change from being brittle to being ductile; The standard deviation was used to quantify the anisotropy degree, as the water content increases, the standard deviation increases from 0.057 to 0.139, indicating a significant increase in anisotropy of the *K*_Ic_ of the mudstone specimens; In addition, the acoustic emission (AE) system was used to detect the AE events associated with the fracture initiation and propagation in the mudstone specimens for the different water content, with the raising water content, the cumulative AE events decrease, and the standard deviation of AE events increases which indicates that the anisotropy of the AE events of the three types of specimens becomes more prominent. The relationship between *σ*_t_ and *K*_Ic_ of the three types of mudstone specimens for different water contents is a linear relation, however, the three fitting curve equations differ from each other, and have some differences with the result of Zhang [32] because of the influence of the bedding structures.
